# Concomitant Thermochromic and Phase‐Change Effect in a Switchable Spin Crossover Material for Efficient Passive Control of Day and Night Temperature Fluctuations

**DOI:** 10.1002/advs.202202253

**Published:** 2022-06-16

**Authors:** Esther Resines‐Urien, Miguel Ángel García García‐Tuñón, Mar García‐Hernández, Jose Alberto Rodríguez‐Velamazán, Ana Espinosa, Jose Sánchez Costa

**Affiliations:** ^1^ IMDEA Nanociencia C/ Faraday 9 Madrid 28049 Spain; ^2^ Instituto de Cerámica y Vidrio CSIC C/Kelsen s/n Madrid 28240 Spain; ^3^ Instituto de Ciencia de Materiales de Madrid CSIC C/Sor Juana Inés de la Cruz, 3 Madrid 28049 Spain; ^4^ Institut Laue‐Langevin 71 avenue des Martyrs, BP 156 Grenoble Cedex 9 38042 France

**Keywords:** buildings, energy, light‐reflection, phase change materials, spin crossover, switchable, thermochromic

## Abstract

The increasing environmental protection demand has prompted the development of passive thermal regulation systems that reduce temperature fluctuations in buildings. Here, it is demonstrated that the heat generated by the sun can trigger a spin crossover (SCO) in a molecule‐base material, resulting in a concomitant color variation (from pink to white) and a phase transition. This leads to a cooling effect with respect to other thermochromic materials. In addition, when the material is cooled, a dampening of the temperature decrease is produced. Therefore, these materials can potentially be implemented for passive temperature control in buildings. Furthermore, SCO materials are remarkably stable upon cycling and highly versatile, which allows for the design of compounds with properties tailored for the desired climatic conditions and comfortable temperature.

## Graphical abstract

The heat produced by the sun can induce a spin transition in a spin‐crossover material, leading to a color change coupled with the absorption/release of energy, which results in a cooling effect at high temperatures and a dampening of the temperature decrease at low temperatures. Hence, these materials can potentially be applied for passive temperature control in buildings.

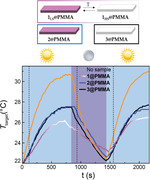

## 1 Introduction

Nowadays, building thermalization is a widespread human necessity, accounting for 30% of the total final energy use and 28% of energy‐related carbon dioxide (CO_2_) worldwide emissions.^[^ ^1^ ^]^ These CO_2_ emissions are a major contributor to climate change, which has become one of the biggest concerns of humankind.^[^ ^2^ ^]^ A reduction in emissions could be achieved by eliminating coal, oil, and natural gas as sources of building heating, and by reducing the energy consumption derived from the use of air conditioning systems.^[^ ^3^ ^]^ Thus, the growing energy‐saving demand and environmental protection have prompted the development and implementation of more energy‐efficient and environmentally friendly thermalization technology. In this regard, remarkable efforts have been focused on the implementation of passive thermal regulation systems, that can be incorporated directly into windows,^[^ ^4^, ^5^, ^6^, ^7^, ^8^, ^9^ ^]^ roofs, or walls of buildings and operate without the need for electricity.^[^ ^10^, ^11^, ^12^ ^]^

Although to keep buildings cool the light absorption and reflection properties have been used for millennia, nowadays more sophisticated approaches are being explored for passive thermal regulation of buildings. The first one is based on the so‐called radiative cooling technique, in which a surface is naturally cooled by reflecting sunlight and radiating heat to the cold outer space.^[^ ^13^, ^14^, ^15^, ^16^, ^17^ ^]^ The second approach uses hygroscopic and/or porous materials to store and release water when heated, leading to evaporative cooling, where heat is dissipated through water evaporation.^[^ ^18^, ^19^, ^20^, ^21^, ^22^ ^]^ The last one is the use of phase‐change materials (PCMs), which present high‐density thermal energy storage, therefore minimizing temperature fluctuations through the use of latent heat.^[^ ^23^, ^24^, ^25^, ^26^, ^27^ ^]^

Employing the first two approaches, subambient cooling was achieved both during the day and night. While this is interesting for places where the weather is hot throughout the whole day, certain regions of the world, with climates where high temperatures are reached during the day and low temperatures at night, for example, New Delhi,^[^ ^28^ ^]^ require different materials to reduce temperature fluctuations in both directions. Therefore, certain PCMs are so far applied for thermal regulation in both senses, although they display some limitations.^[^ ^29^, ^30^ ^]^ Nevertheless, from a global warming perspective, the need to reduce temperature fluctuations calls for the development of new materials and/or novel alternative approaches to tackle this problem in a more effective manner.

In this respect, a new strategy could be the use of materials that combine two of the principles mentioned before by switching between multiple phases that display distinct thermo‐induced optical properties, resulting in different light reflections,^[^ ^31^, ^32^, ^33^ ^]^ coupled with the release/absorption of energy associated with a phase transition. One can easily imagine the advantage in thermal efficiency for buildings with a coating that changes color from a clear, reflecting color in summer to a darker, absorbing color in winter, or even in shorter day/night cycles since they could also dampen thermal fluctuations as a consequence of the phase change.

**Figure 1:**
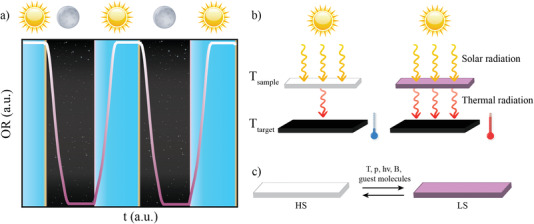
a) Scheme of the behavior of a SCO material during day‐night cycles. b) Working scheme of a SCO material under solar radiation. When the weather is hot, and the material is in its HS state (white), the light is reflected and the increase in the temperature of the room is controlled. On the contrary, under cold temperatures, the materials show the LS state (pink), so more wavelengths are absorbed and the room is heated. c) Illustration of the spin transition under external stimuli.

A new approach could be the use of molecule‐based switchable spin crossover (SCO) compounds.^[^ ^34^, ^35^, ^36^, ^37^, ^38^, ^39^, ^40^ ^]^ These materials undergo a spin transition between two electronic states, low spin (LS) and high spin (HS) upon external stimuli, including temperature variation. This spin transition is associated with a color change,^[^ ^41^ ^]^ which could result in a different capacity for reflecting sunlight (see **Figure** **1**).^[^ ^42^, ^43^ ^]^ In addition, the shift from LS to HS is an endothermic process, so the SCO material absorbs heat. Correspondingly, when the temperature decreases, the material changes from HS to LS in an exothermic process, where the material desorbs heat, contributing to reducing the temperature drop in a room.

The application of SCO materials for thermal management purposes has been considered recently in different contexts. In particular, there have been a few reports about possible barocaloric refrigeration applications^[^ ^44^, ^45^, ^46^ ^]^ as well as the use of SCO materials as thermal protective barriers in microelectronic circuits^[^ ^47^, ^48^ ^]^ and in buildings.^[^ ^49^ ^]^ However, these ideas consisted of the use of latent heat associated with the SCO, in contrast to the present work, in which we consider primarily the role of thermochromic properties. The key advantage in the use of these switchable materials is that the appropriate selection of a SCO material could result in effective passive temperature control in circumstances where large temperature fluctuations need to be avoided (unlike reflecting coatings, which are only beneficial to reduce warming during sunlight exposure). Moreover, the remarkable versatility of SCO compounds may allow adapting the desired phase change temperature and hysteresis to the climatic conditions and the desired comfort temperature. Therefore, the objective of this work is to give a proof‐of‐concept of this idea by demonstrating, in the first place, that the heat generated by the solar radiation is enough to produce a spin transition in an SCO material and, second, that the use of this material allows, in both heating and cooling, to reduce temperature fluctuations.

## 2 Results and Discussion

Here, three different molecular‐based coordination polymers [Fe(NH_2_trz)_3_](OTs)_2_ (1), [Fe(Htrz)_2_(trz)[(BF_4_) (2) and [Zn(NH_2_trz)_3_](NO_3_)_2_ (3) have been synthesized, where NH_2_trz = 4‐Amino‐4H‐1,2,4‐triazole; OTs = *p*‐toluenesulfonate; Htrz = 1,2,4‐Triazole and trz = 1,2,4‐triazolate. These materials present the widely studied structure of the family of the triazole‐based SCO coordination polymers (Figure S3, Supporting Information).^[^ ^50^, ^51^, ^52^ ^]^ That is to say, the metal center is in an octahedral environment, coordinated by six nitrogen atoms from six different triazole units. The triazoles act as bridging ligands between two metal centers, leading to the formation of 1D chains. When Fe(II) is used as the metal center, these materials can exhibit SCO properties. Of particular interest for this work is that compound 1 displays this spin transition around room temperature (RT). To investigate and compare the temperature control ability of 1, two different compounds (Figure S2, Supporting Information) were synthesized: a compound that is only in its LS state in this temperature range (2), and thus remains pink, and another that is white (3).

**Figure 2:**
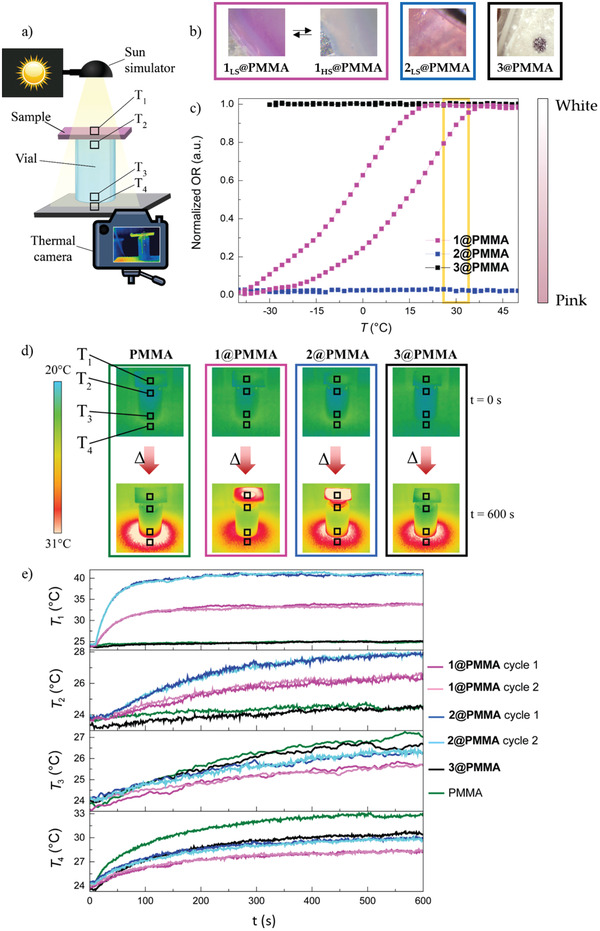
a) Scheme of the experimental setup. b) Images of the composites. c) Optical reflectivity measurements for **1@PMMA**‐**3@PMMA**. The temperature region encompassed by these sunlight exposure measurements is marked in yellow. The optical reflectivity has been normalized with respect to **1@PMMA**. d) Thermal images for **1@PMMA**‐**3@PMMA** and PMMA at times 0 and 600 s under sunlight exposure. The measured area in each sample is marked with a square in the images. e) Temperature vs exposure time (the sun simulator is turned on after the first 10 s) at the four measured spots.

To facilitate the use of these powder‐like materials, compounds 1–3 were embedded^[^ ^53^, ^54^ ^]^ in organic polymer polymethyl methacrylate (PMMA), from here on 1@PMMA, 2@PMMA, and 3@PMMA, respectively (**Figure** **2**b). Optical and magnetic SCO properties were explored by optical reflectivity (OR) and SQUID magnetometry, both in the coordination polymers and when combined with PMMA (Figure 2c and Figures S3–S9, Supporting Information). As expected, the spin transition properties of 1@PMMA and 2@PMMA compared to 1 and 2 are maintained, with the only difference being that the hybrid materials show a gradual transition compared to the powder samples (see Figures S3–S9, Supporting Information for further details). Importantly, this thermochromic process is reversible and can be maintained for several thermal cycles (see Figure S10, Supporting Information for 40 heating‐cooling cycles).^[^ ^55^ ^]^ Naturally, neither compound 3 nor 3@PMMA displays a spin transition, since Zn(II) is a d^10^ transition metal, and the possibility of SCO is nonexistent. All these materials were further characterized by Fourier‐transform infrared spectroscopy (FTIR), powder X‐ray diffraction (PXRD), and thermal gravimetric analysis (TGA) (see Figures S11–S21, Supporting Information, for further details). These techniques confirm that the desired reported compounds have been obtained.^[^ ^56^, ^57^ ^]^ The absorption spectra for 1@PMMA‐3@PMMA, and PMMA (Figures S22–S25, Supporting Information) were measured in the temperature range where the experiment takes place. The spectra remain constant with temperature for 2@PMMA, 3@PMMA, and PMMA, while clear differences can be observed for 1@PMMA. In this case, as the temperature increases, and the composite becomes light pink, the light absorption decreases. This is in agreement with the optical reflectivity measurements.

In order to simulate temperature fluctuations in a room, two different experiments were devised. The first one (Figure 2a; Figure S26, Supporting Information) consists on placing the polymers (1@PMMA‐3@PMMA and PMMA) on top of an empty glass vial under the irradiation of a solar lamp (sun simulator, 1000 W m^–2^). This configuration mimics the temperature in a closed room. This process was recorded with an infrared thermal camera, so that the temperature inside the vials and in the polymer could be characterized over time, visualizing their thermal gradients.

The recorded infrared thermal images can be seen in Figure 2d, at times 0 and 600 s from the start of sunlight irradiation. Four different spots have been measured in relation to the irradiation time (Figure 2e): the sample temperature (T_1_), a temperature inside the vial (T_2_), and two temperatures underneath the vial: one inside the vial (T_3_) and another outside (T_4_).

The sample temperature (T_1_, top graph) shows that 2@PMMA increases from 24 to 41.6 °C (Δ*T* = 17.6 °C). Therefore, starting from roughly the same temperature, the 2@PMMA temperature increases 8 °C more than that of 1@PMMA (Δ*T* = 10 °C), and 16 °C more than in 3@PMMA and PMMA (Δ*T* = 1.3 °C). The rate at which the temperature increases in the first 2 min is as follows: 1@PMMA 4 °C min^−1^, 2@PMMA 8 °C min^−1^, 3@PMMA 0.5 °C min^−1^, and PMMA 0.4°C min^−1^. Upon examination of T_2_, it can be seen that in all cases, an increase in temperature is observed as the irradiation time progresses, although in 3@PMMA and PMMA the temperature rises significantly less than in the other two. For compounds 1@PMMA and 2@PMMA, the temperature increases with time in a similar manner, up to 50 s, where the two plots diverge and 2@PMMA heats up more than the SCO sample. More precisely, the temperature in 2@PMMA increases by 14.4%, and by 10.2% for 1@PMMA, which means a slight control in temperature was achieved.

On the other hand, for T_3_ the greatest thermal increment is observed for 3@PMMA and PMMA. This indicates that the sunlight is able to pass through the white materials and raise the temperature inside the room. 2@PMMA follows as the next sample that reaches the highest temperature on the floor. Therefore, the tendency: PMMA >3@PMMA >2@PMMA >1@PMMA can be explained by a synergy occurring between two different effects. The first is that the completely white samples do not heat up, but allow solar radiation to pass through and, consequently, the temperature in the room increases.

The opposite effect is observed in the case of 2@PMMA, where the material itself is the one that heats up the most and results in a temperature increase. This is because dark materials absorb more wavelengths of light and convert them into heat. It is important to note that 1@PMMA is in a bistable region, so it is neither completely white nor as pink as it would be when it is completely in LS. It therefore shows a synergy of these two effects: it starts with a slightly pink color, which impedes solar radiation from passing through the material, and becomes almost white, allowing it to absorb with less intensity. This same behavior can be observed for T_4_. To confirm that this behavior can be replicated, the 1@PMMA and 2@PMMA materials were measured twice. In between the measurements, the samples were cooled, so that the materials recover their initial state. For both materials and in the four measured spots, it is evident that the temperature dependence with time is replicable.

The different behaviors between 1@PMMA and 2@PMMA can be explained by plotting the temperature in the 1@PMMA polymer (Figure 2e, upper graph). At the beginning of the measurement, the 1@PMMA film is at 23.8 °C, which according to the optical reflectivity measurements (Figure 2c), corresponds to a 74% of HS, while by the end of the measurement, the temperature of the composite reaches a maximum of 33.9 °C, where 95% of the iron centers in the composite are in HS. This indicates that the heat generated by the sun simulator (or the sun itself) is enough to provoke a partial spin transition in the material. As previously discussed, the spin transition from LS to HS results in a color change from pink (LS) to white (HS). Although the spin transition is only partial, a fraction of 21% of the iron(II) ions changed from LS to HS in this temperature range, which is sufficient for a color change to be seen by the naked eye (Figure S27, Supporting Information). Thus, the material is able to dissipate heat more efficiently than the analogous pink sample 2@PMMA in which the SCO does not occur in this temperature range.

**Figure 3:**
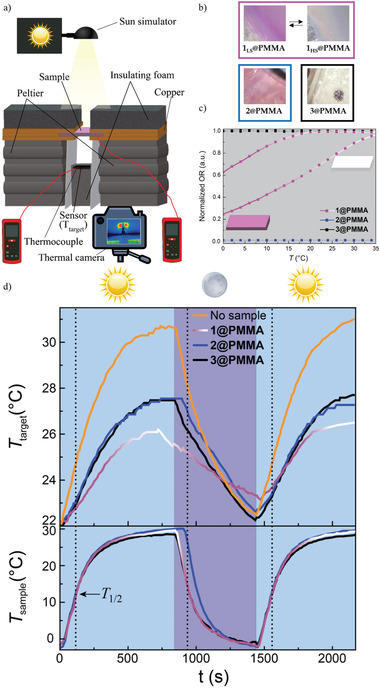
a) Scheme of the experimental setup. b) Images of the composites at the highest and lowest measured temperature. c) Optical reflectivity measurements for **1@PMMA**‐**3@PMMA** in the temperature region encompassed by these sunlight exposure measurements. The optical reflectivity has been normalized in respect to **1@PMMA**. d) Temperature vs exposure time with on‐off solar simulator cycles.

The second setup (**Figure** **3**a; Figure S28, Supporting Information) was designed to characterize hot‐cold cycles and avoid contributions from conduction and convection (due to sample contact with the vial). Here, the sample is placed between copper foils, that are in contact with two Peltiers (see setup description in the Supporting Information). This enables the sample to be cooled below RT. The polymer is irradiated with the solar lamp, and the temperature fluctuations are measured using an anodized aluminum sensor, placed underneath the sample (*T*_target_). The composite temperature is recorded with an infrared thermal camera, and the sensor temperature using a thermocouple.

In Figure 3d, the samples are subjected to hot‐cold cycles, simulating day and night conditions. Before starting the experiment, the solar lamp is off and the two Peltier are on, to bring the samples at the same temperature (Figure S29, Supporting Information) to perform comparable hot‐cold cycles. Afterward, the solar simulator is turned on and the Peltier off, which corresponds to the heating process. The two temperatures *T*_sample_ and the *T*_target_ were followed vs the time. The *T*_sample_ increases following this sequence: 2@PMMA >1@PMMA >3@PMMA (Figure S29, Supporting Information). However, *T*_target_ rises following this 3@PMMA >2@PMMA >1@PMMA order. When the experiment is carried out without the presence of a sample, *T*_target_ increases rapidly, reaching the highest temperature.

The cooling cycle starts at time 800 s, when the solar lamp is switched off and the Peltier on (dark blue background). Immediately, both *T*_sample_ and *T*_target_ begin to drop. *T*_target_ reaches a minimum temperature at 1480 s. Interestingly, this minimum is higher for 1@PMMA with respect to the references 2@PMMA and 3@PMMA, and higher than when no sample is employed.

Again, the system is set in heating mode and the same behavior as in the first cycle is observed: 2@PMMA and 3@PMMA reach higher temperatures than 1@PMMA and the highest temperature is reached when no sample is used. These experiments were repeated, and the same result was obtained (Figure S29, Supporting Information).

From the sample temperature, it can be stated that 1@PMMA is shifting from 22% of HS Fe(II) ions (at −2 °C) to 88% of HS at the highest temperature (29.6 °C). This translates into fewer temperature fluctuations during both cooling and heating. The control at high temperatures can be associated with the synergy between thermochromism and phase change. The control in the low‐temperature region is believed to be due to the energy release associated with the spin transition, with typical Δ*H*_LS‐HS_ values in the range of 50 kJ mol^−1^.^[^ ^58^ ^]^ While the Δ*H*_PCM_ reported values are generally higher (Table S1, Supporting Information),^[^ ^23^, ^26^, ^59^, ^60^ ^]^ the combination of both the thermochromic properties and the enthalpy associated with the phase change could be a groundbreaking approach to reducing temperature fluctuations in both directions.

In addition, to simulate what would occur upon a temperature decrease under solar irradiation, this same setup was used but, in this case, the solar lamp remains lit during the entire experiment, accompanied by Peltier on‐off cycles (Figure S30, Supporting Information). At the beginning of the experiment, the Peltier is switched on, resulting in a cooling of the sample, and to some extent, also of the sensor. The main observation here is that the minimum recorded *T*_target_ (25 °C) is reached more slowly for 1@PMMA when in comparison with the references (at times 400, 750, and 910 s, for 2@PMMA, 3@PMMA, and 1@PMMA, respectively). When no sample is used, the minimum *T*_target_ recorded is 29 °C. This result reinforces the conclusion that the material exhibiting SCO is capable of reducing temperature fluctuations and providing certain control over the temperature.

## 3 Conclusion

To summarize, this work demonstrates that the heat generated by the sun is sufficient to produce, at least, a partial spin transition in an SCO material. This, in turn, leads to a cooling effect with respect to other similar materials, due to an increase in light reflection resulting from the color change and the energy absorption associated with the spin transition. In addition, when the material is cooled to temperatures easily attainable at night (−2 °C), a dampening of the temperature decrease is produced. This is believed to be due to the energy release associated with the spin transition. Here, the material also regains its initial behavior and can be employed again to control fluctuations at increasing temperatures. Therefore, this type of material can be used to reduce temperature fluctuations, which could potentially be implemented for passive temperature control in buildings, by combining selective light reflection and phase‐change materials. SCO molecule‐based materials are remarkably stable upon cycling and highly versatile, which may allow for the design of compounds adapting the intended properties (transition temperature and hysteresis) for the climatic conditions and the desired comfort temperature.

## 4 Experimental Section

Compound 1 was synthesized at room temperature, by adding drop by drop to a solution of 0.59 mmol of NH_2_trz (NH_2_trz = 4‐Amino‐4H‐1,2,4‐triazole) in 3 mL of ethanol on top of a solution of 0.20 mmol of Fe(OTs)_2_ (OTs = tosylate) in 3 mL of distilled water. The solution was stirred for 1 h, filtered, and washed with ethanol. 1 was obtained as a pink powder in 87% yield.

Anal. calcd for 1 0.4H_2_O: C 36.53%, H 4.11%, N 25.56%; found C 36.34%, H 4.05%, N 25.79%.

FTIR 1 (cm^−1^): *ν* = 3441 (w; *ν*(OH)), 3293 (m; *ν*(NH)), 3210 (w), 3062 (m; *ν*(CH)_ar_), 3012 (w), 2924 (w), 1631 (m), 1600 (w), 1546 (m; *δ*(NH)), 1496 (w), 1449 (w; *δ*(CH)_ar_), 1396 (w), 1170 (s; *ν*(S = O)_OTs_), 1122 (s), 1098 (m), 1033 (s; *ν*(S = O)_OTs_), 1008 (s; *ν*(S = O)_OTs_), 908 (w), 881 (w), 813 (m; *δ*(ring)), 709 (w), 681 (s; *ν*(CS)_OTs_), 623 (s), 563 (s; *ν*(CS)_OTs_), 493 (w), 457 (w).

Compound 2 was synthesized at room temperature, dissolving 1 mmol of Fe(BF_4_)_2_ 6H_2_O in 3 mL of distilled water and adding it drop by drop to a solution of 3 mmol of Htrz (Htrz = 1,2,4‐triazole) in 3 mL of ethanol. The resulting solution was stirred for 24 h, filtered, and washed with ethanol obtaining 2 as a pink powder (76% yield).

Anal. calcd for 2 1.35 H_2_O: C 19.31, H 2.89, N 33.78; found C 19.07, H 2.66, N 33.79.

FTIR 2 (cm^−1^): *ν* = 3170 (w; *ν*(NH)), 3093 (w; *ν*(CH)_ar_), 3008 (w), 2915 (w), 2845 (w), 2698 (w), 2621 (w), 2535 (w), 2535 (w), 2457 (w), 1749 (w), 1719 (w),1639 (w), 1535 (m; *δ*(NH)), 1495 (m), 1453 (m; *δ*(CH)_ar_), 1309 (m), 1285(m), 1220 (w), 1190 (w), 1163 (m), 1144 (m), 1063 (s; *ν*(BF)_BF4_), 1034 (s; *ν*(BF)_BF4_), 977 (m), 912 (w), 866 (m), 827 (w), 765 (w), 679 (m), 631 (s), 523 (m), 468 (w), 437(w).

Compound 3 was synthetized at room temperature, by adding drop by drop to a solution of 3 mmol of NH_2_trz (NH_2_trz = 4‐Amino‐4H‐1,2,4‐triazole) in 3 mL of ethanol on top of a solution of 1 mmol of Zn(NO_3_)_2_ in 3 mL of distilled water. The solution was stirred for 1 h, and the white precipitate was filtered and washed with ethanol. 3 was obtained as a white powder (85% yield).

Anal. calcd for 3·1H_2_O: C 15.68, H 3.07, N 42.66; found C 15.33, H 3.14, N 43.01.

FTIR 3 (cm^−1^): *ν* = 3520 (w), 3319 (m; *ν*(NH)), 3218 (w), 3070 (m; *ν*(CH)_ar_), 3004 (w), 1753 (w), 1632 (m; *ν*(NO)_as,NO3_), 1547 (m; *δ*(NH)), 1485 (w; *δ*(CH)_ar_), 1339 (s; *ν*(NO)_sim,NO3_), 1219 (m), 1088 (m), 1030 (m), 996 (m), 904 (w), 828 (m; *ν*(NO)_trans/cis,NO3_), 712 (w), 690 (w), 622 (s), 421 (w).

The 1@PMMA, 2@PMMA, and 3@PMMA films (3% w/w) were prepared by mixing 0.2 g of the respective sample powder with 1.98 g of PMMA (PMMA = poly(methyl methacrylate; average M_w_ ≈120 000 g mol^−1^). This mixture was dissolved in CHCl_3_, sonicated for 30 min, and deposited in a mold (5 cm × 5 cm), that was placed in the fridge overnight so the CHCl_3_ evaporates slowly and no bubbles appear. The composite was then carefully extracted from the mold.

FTIR 1@PMMA (cm^−1^): *ν* = 3290 (w; *ν*(NH)), 3110 (w; *ν*(CH)_ar_), 2995 (w; PMMA), 2950 (m; PMMA), 2851 (w), 1724 (s; PMMA), 1614 (w), 1546 (w; *δ*(NH)), 1480 (w; PMMA), 1446 (w; PMMA), 1435 (m), 1386 (w; PMMA), 1269 (w; PMMA), 1239 (w; PMMA), 1189 (w; PMMA), 1169 (w; *ν*(S = O)_OTs_), 1146 (s; PMMA), 1122 (s), 1098 (w), 1033 (m; *ν*(S = O)_OTs_), 1009 (m; *ν*(S = O)_OTs_), 988 (w; PMMA), 966 (w; PMMA), 912 (w; PMMA), 880 (w), 841 (w; PMMA), 812 (m; *δ*(ring), 749 (s; PMMA), 683 (s; *ν*(CS)_OTs_), 625 (s), 563 (s; *ν*(CS)_OTs_), 481 (w; PMMA).

FTIR 2@PMMA (cm^−1^): *ν* = 3170 (m; *ν*(NH)), 3095 (w; *ν*(CH)_ar_), 3006 (m), 2915 (m), 2848 (m), 2748 (w), 2698 (w), 2622 (w), 2536 (w), 1728 (m; PMMA), 1644 (w), 1536 (m; *δ*(NH)), 1496 (m), 1453 (m), 1388 (w), 1309 (m), 1284 (w), 1221 (w; PMMA), 1190 (w; PMMA), 1162 (m), 1144 (m), 1109 (w), 1063 (s; *ν*(BF)_BF4_), 1034 (s; *ν*(BF)_BF4_), 977 (m), 912 (w), 866 (m), 826 (w; PMMA), 764 (s; PMMA), 679 (m), 631 (s), 524 (m), 482 (w; PMMA), 471 (w), 437 (w).

It should be noted that at temperatures above 125 °C the PMMA started degrading, so 2@PMMA, whichundergoes the spin transition at higher temperatures, could not be characterized in HS by SQUID and only partially by optical reflectivity. FTIR 3@PMMA (cm^−1^): *ν* = 3321 (w; *ν*(NH)), 3219 (w), 3070 (w; *ν*(CH)_ar_), 2998 (m; PMMA), 2950 (m; PMMA), 2844 (w), 1721 (s; PMMA), 1635 (w; *ν*(NO)_as,NO3_), 1547 (w; *δ*(NH)), 1481 (w; PMMA), 1434 (m; PMMA), 1363 (m; *ν*(NO)_sim,NO3_), 1269 (w; PMMA), 1238 (m; PMMA), 1190 (w; PMMA), 1143 (s; PMMA), 1088 (m), 1062 (w; PMMA), 1029 (w), 993 (m), 968 (w; PMMA), 913 (w), 842 (w; PMMA), 828 (w; *ν*(NO)_trans/cis,NO3_), 748 (s; PMMA), 667 (m; PMMA), 623 (m), 554 (w; PMMA), 479 (w; PMMA), 458 (w).

Optical reflectivity measurements performed using a MOTIC SMZ‐171 optical stereoscope coupled with a MOTICAM 3. Images were collected in BMP format without any filter using the Motic Images Plus 3.0 software, with the mean value from each region of interest (ROI) analyzed under the ImageJ program. The temperature was controlled using a Linkam T95 system controller and a LNP 95 Liquid Nitrogen Cooling System.

Thermogravimetric analyses performed using a TA Instrument TGAQ500 with a ramp of 2 °C min^−1^ under air from 30 to 600 °C.

FT‐IR spectra recorded as neat samples in the range 400–4000 cm^−1^ on a Bruker Tensor 27 (ATR device) Spectrometer.

Elemental analyses were performed on a LECO CHNS‐932 Analyzer at the “Servicio Interdepartamental de Investigación (SIdI)” at Autónoma University of Madrid.

Magnetic susceptibility measurements carried out in a Quantum Design MPMS‐5S SQUID magnetometer under a 10 000 Oe field at a rate of 1 °C min^−1^. Each sample was secured inside a plastic capsule. Pascal constants were used to correct the diamagnetic contribution.

Powder X‐ray diffraction collected in a Rigaku Smartlab SE diffractometer with a Bragg‐Brentano configuration, using Cu‐K*α* radiation (*λ* = 0.1541 nm). The samples were measured between 5° and 50° with a speed of 1.8° min^−1^ under an X‐Ray fluorescence reduction mode, at room temperature.

Absorption spectra measured with a CCS200 – Compact Spectrometer between 1000–400 cm^−1^. The temperature dependence experiments were performed using the Linkam T95 system controller and a LNP 95 Liquid Nitrogen Cooling System.

Thermo‐optic analyses performed using a VeraSol LED solar simulator (Newport) producing 1 sun AM 1G (1000 W m^−2^) simulated sunlight.

Thermal images acquired with a real‐time infrared camera FLIR T540 (FLIR Ltd, USA) (30 mK thermal sensitivity and 464 × 348 pixels resolution). Measurements were performed several times to ensure reproducibility. Each measurement was conducted for 10 min till thermal equilibrium (or steady‐state temperature) was reached ±2% of the reading.

A RS41 Digital Thermometer (±0.5% of reading) was employed to measure the target temperature in the second experimental setup.

## Conflict of Interest

The authors declare no conflict of interest.

## Supporting information

Supporting Information

## Data Availability

The data that support the findings of this study are available in the supplementary material of this article.
