# Supplementary material for: Concomitant Thermochromic and Phase‐Change Effect in a Switchable Spin Crossover Material for Efficient Passive Control of Day and Night Temperature Fluctuations
Source: Adv Sci (Weinh). 2022 Jun 16;9(24):2202253. doi: 10.1002/advs.202202253 (PMC9404398; doi:10.1002/advs.202202253)
Supplement: Supplementary file 1 — Supporting Information [file ADVS-9-2202253-s001.pdf]

Supporting Information

Concomitant thermochromic and phase-change effect in a switchable spin crossover material for efficient passive control of day and night temperature fluctuations

Esther Resines-Urien, Miguel Ángel García García-Tuñón, Mar Garcia-Hernandez, J. Alberto Rodríguez-Velamazán, Ana Espinosa, Jose Sanchez Costa**

1) Polymeric structure
2) Pictures of 1-3
3) Optical reflectivity measurements
4) Magnetic characterization
5) Thermochromic reversibility
6) FTIR
7) Powder X-ray Diffraction (PXRD) study
8) Thermogravimetric analyses
9) Absorption spectra
10) Setup images
11) Second experimental setup
12) Enthalpy values of different phase-change materials

1) Polymeric structure

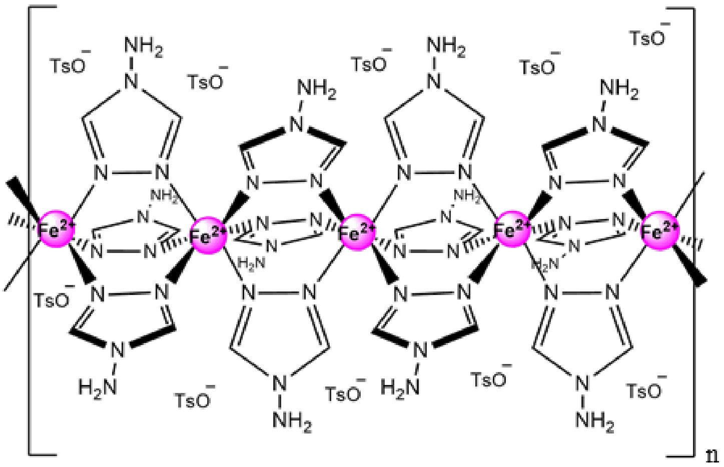

Figure S1. Illustration of the 1D structure of **1**. Compounds **2** and **3** present this same general backbone.

2) Pictures of 1-3

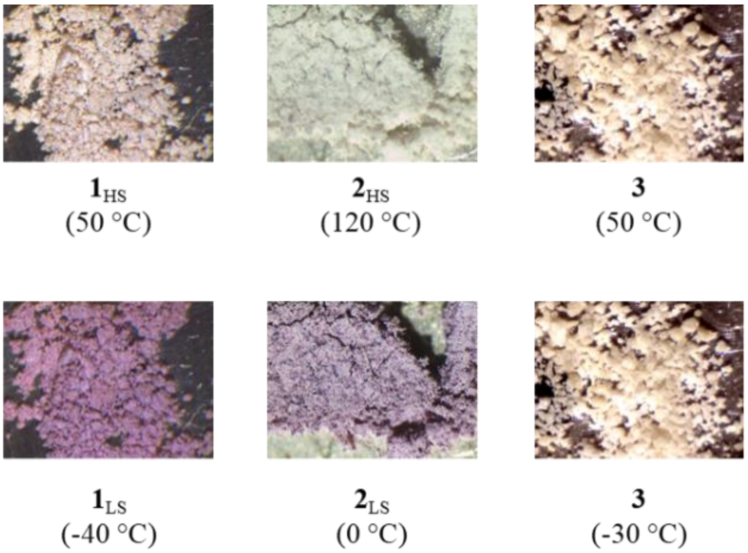

Figure S2. Pictures of **1-3** at the highest and lowest measured temperature, associated with the spin state.

3) Optical reflectivity measurements

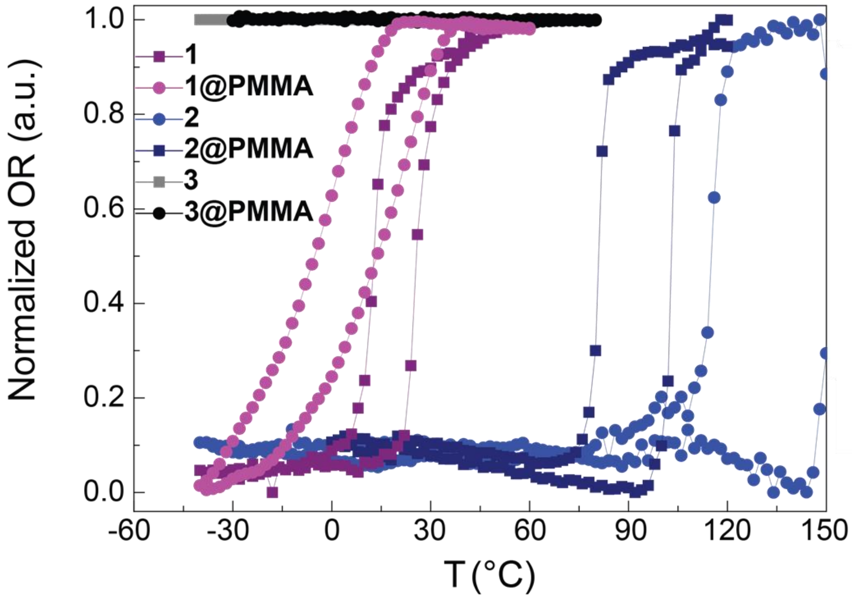

Figure S3. Optical reflectivity measurements for **1-3** and **1@PMMA-3@PMMA**.

4) Magnetic characterization

Compound **1** presents the HS state above RT, and changes abruptly to LS state upon cooling down. When the sample is heated, the HS state is recovered with a hysteresis of 14°C ($T_{1/2}\downarrow$= 12 °C; $T_{1/2}\uparrow$= 26 °C). The same type of behavior is observed for **1@PMMA** although with a much more gradual transition (hysteresis 19 °C; $T_{1/2}\downarrow$= -5 °C; $T_{1/2}\uparrow$= 14 °C). Compound **2** also exhibits a hysteretic SCO behavior between 70 °C and 103 °C. This transition is shifted to even higher temperatures when combined with PMMA ($T_{1/2}\downarrow$= 115 °C). Naturally, neither compound **3** nor **3@PMMA** display a spin transition, since Zn(II) is a d^{10} transition metal, and the possibility of SCO is nonexistent.

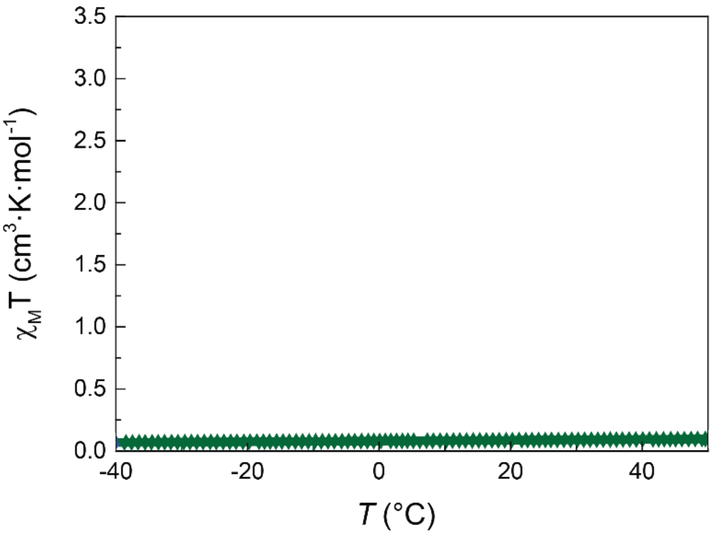

Figure S4. $\chi_M T$ measured as a function of the temperature for **PMMA**.

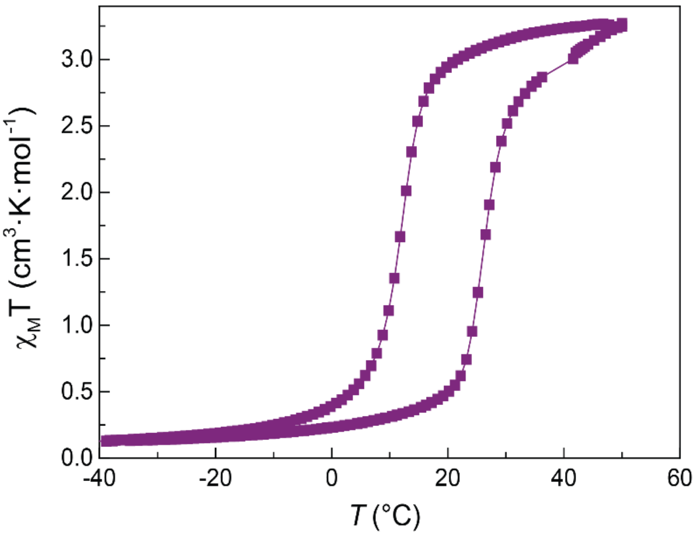

Figure S5. $\chi_M T$ measured as a function of the temperature for **1**.

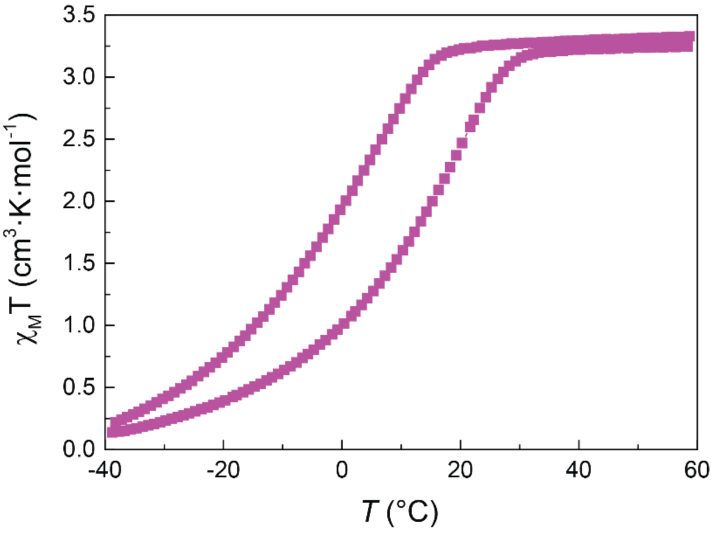

Figure S6. $\chi_M T$ measured as a function of the temperature for **1@PMMA**.

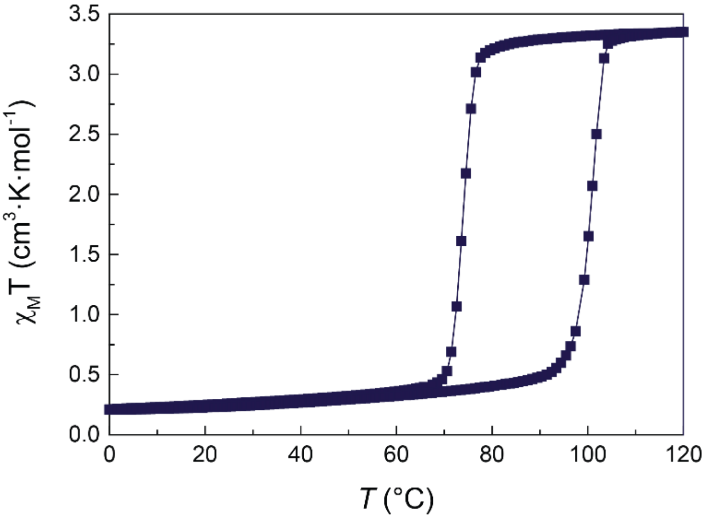

Figure S7. $\chi_M T$ measured as a function of the temperature for **2**.

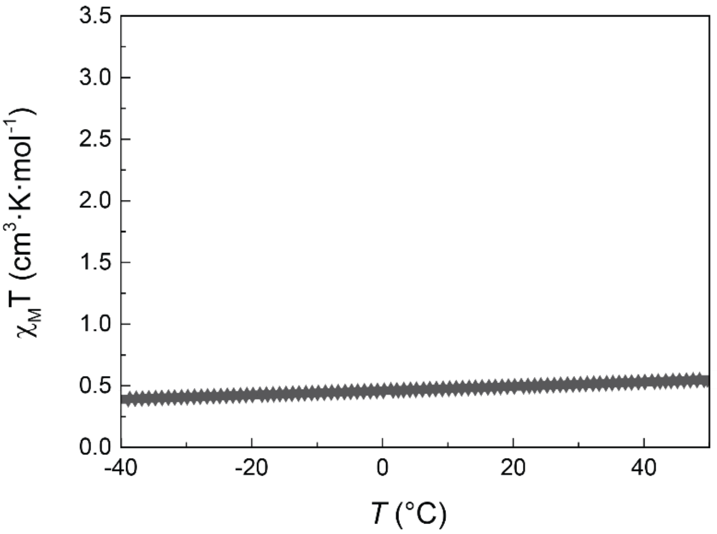

Figure S8. $\chi_M T$ measured as a function of the temperature for **3**.

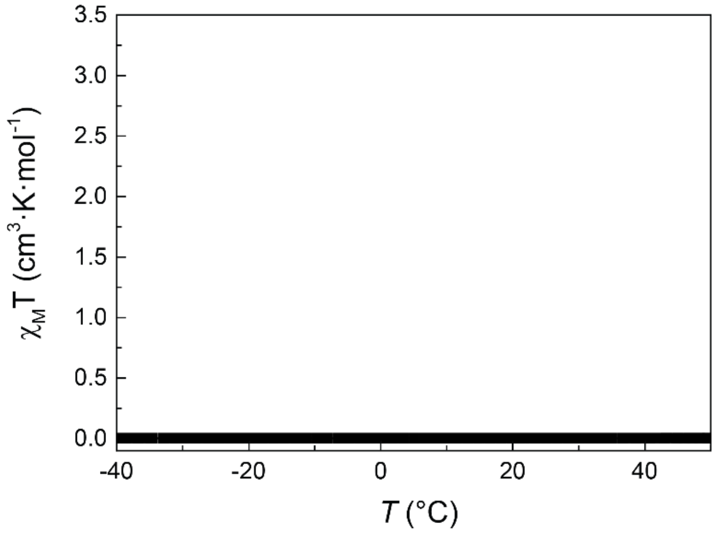

Figure S9. $\chi_M T$ measured as a function of the temperature for **3@PMMA**.

5) Thermocromic reversibility

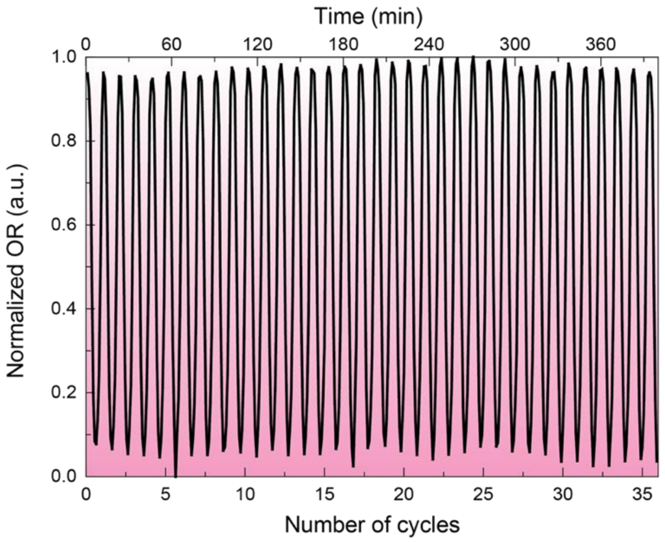

Figure S10. Thermochromic reversibility of **1@PMMA** between -50 °C and 60 °C. The color of the background is associated with the color of the composite: white at high temperatures (HS) and pink at low temperatures (LS).

6) FTIR

The distinctive signals from the coordination polymers and the ones from PMMA can be seen in the **1@PMMA- 3@PMMA** spectra by FTIR spectroscopy.

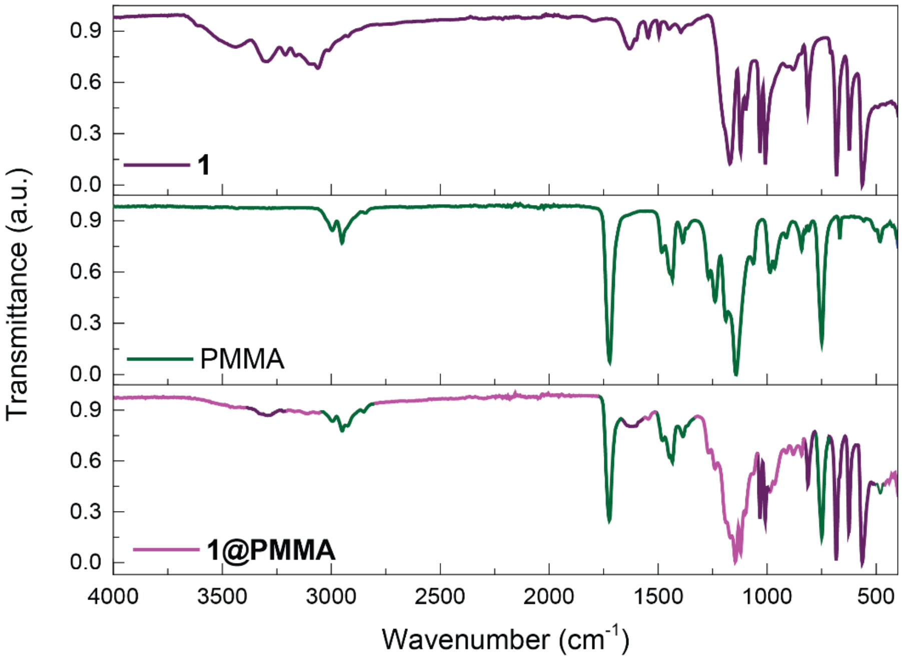

Figure S11. IR spectrum of **1**, PMMA and **1@PMMA** between 4000 cm^{-1} and 400 cm^{-1}. For **1@PMMA** the vibration bands that can be associated with compound **1** are represented in purple, while the ones coming from PMMA are colored in blue. The bands in pink are the result of the combination of **1** and PMMA signals.

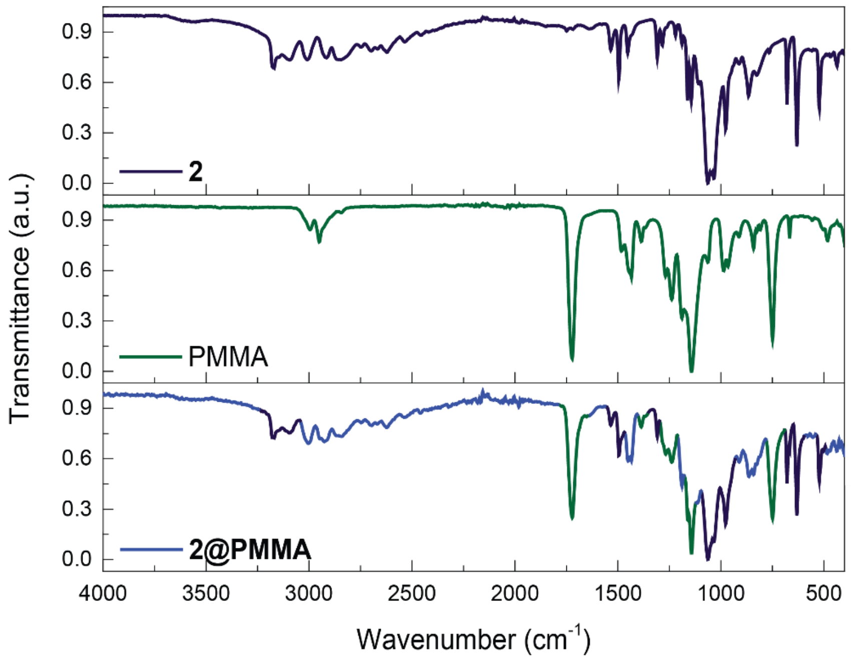

Figure S12. IR spectrum of **2**, PMMA and **2@PMMA** between 4000 cm^{-1} and 400 cm^{-1}. In the case of **2@PMMA** the signals that can be attributed with **2** are depicted in brown, whereas those from PMMA are in blue. The black bands result from the combined signals of **2** and PMMA.

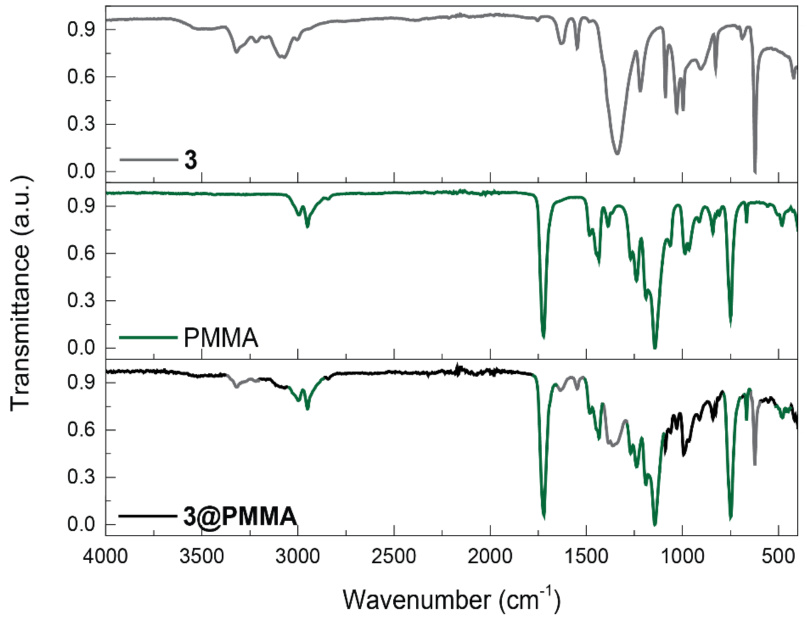

Figure S13. IR spectrum of **3**, PMMA and **3@PMMA** between 4000 cm^{-1} and 400 cm^{-1}. In the spectrum of **3@PMMA** the signals attributable to **3** are shown in maroon, and those of PMMA are indicated in blue. The signals that arise from a combination of **3** and PMMA are in red.

7) Powder X-ray Diffraction (PXRD) study

Even though single crystals of **1-3** could not be obtained, the polycrystalline samples were measured by powder X-ray diffraction PXRD.

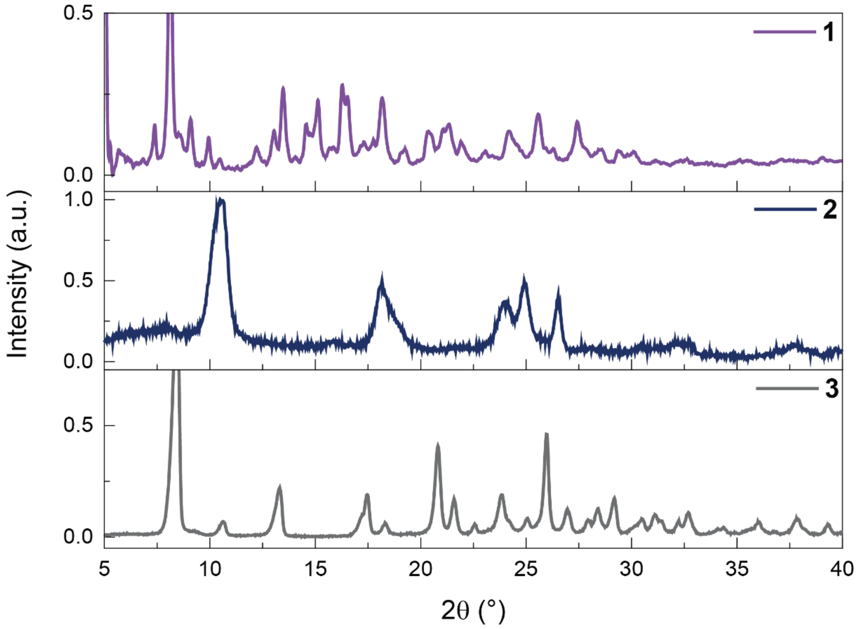

Figure S14. PXRD pattern of **1**, **2** and **3**.

8) Thermogravimetric analyses

The thermogravimetric analyses (TGA) also show that the profiles are different for **1-3**. The profile of **1@PMMA-3@PMMA** in the temperature range between 30 °C and 240 °C is consistent with that of PMMA, with a weight loss between 125 °C and 200 °C. At temperatures higher than 240 °C the weight loss curve follows a similar pattern to that of the corresponding coordination polymer. Hence, **1@PMMA** presents the most gradual weight loss above 240 °C, and a slight plateau can be seen at 300 °C, similarly to **1**. On the other hand, **2** and **2@PMMA** exhibit a one-step abrupt weight loss. Compounds **3** and **3@PMMA** display the most abrupt weight loss, and a plateau can be observed at 300 °C for **3**. While this plateau is not evident in **3@PMMA**, a change in the slope appears at this temperature.

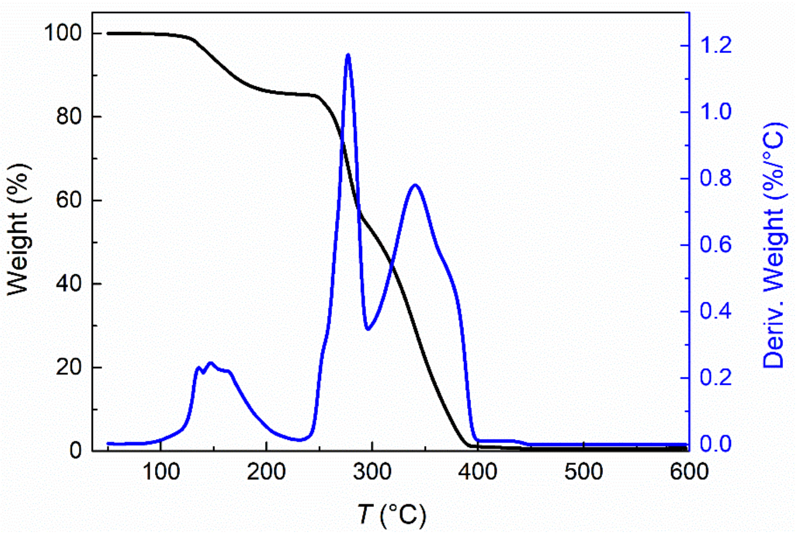

Figure S15. Thermogravimetric analysis of **PMMA** between 30 °C and 600°C.

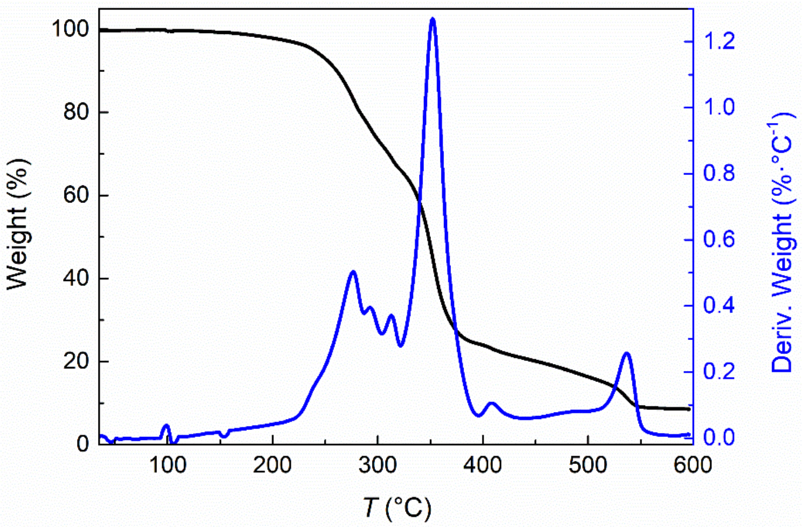

Figure S16. Thermogravimetric analysis of **1** between 30 °C and 600°C.

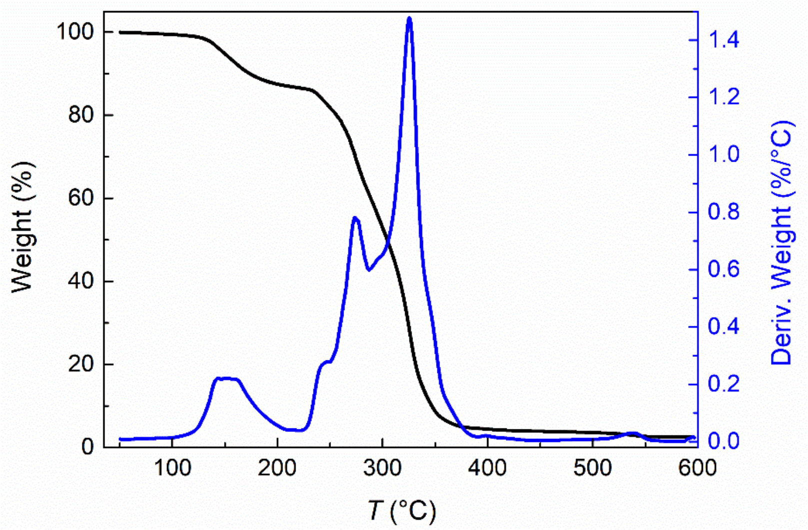

Figure S17. Thermogravimetric analysis of **1@PMMA** between 30 °C and 600°C.

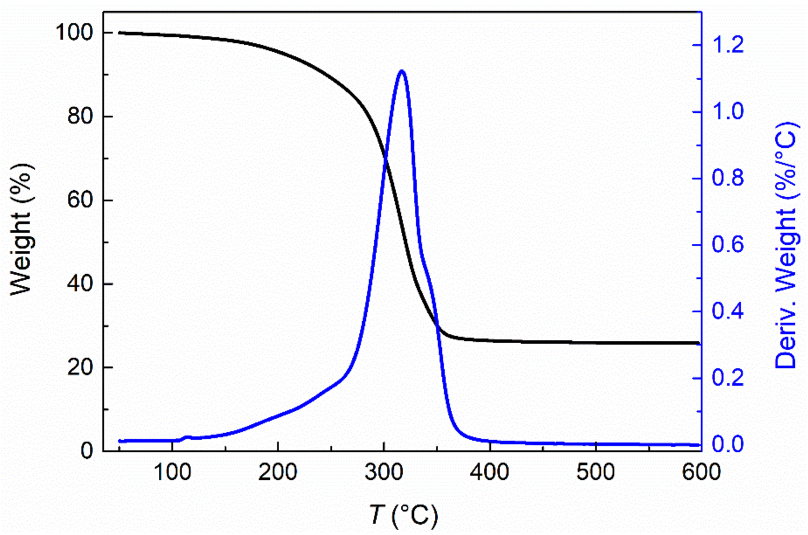

Figure S18. Thermogravimetric analysis of **2** between 30 °C and 600°C.

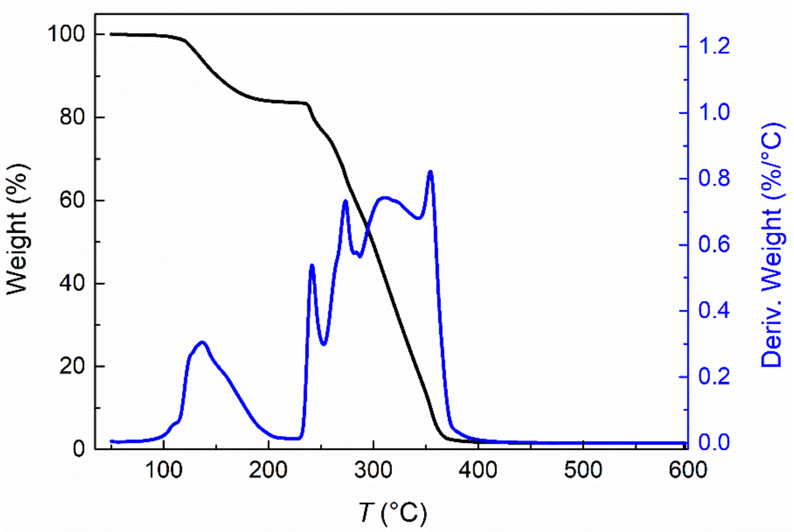

Figure S19. Thermogravimetric analysis of **2@PMMA** between 30 °C and 600°C.

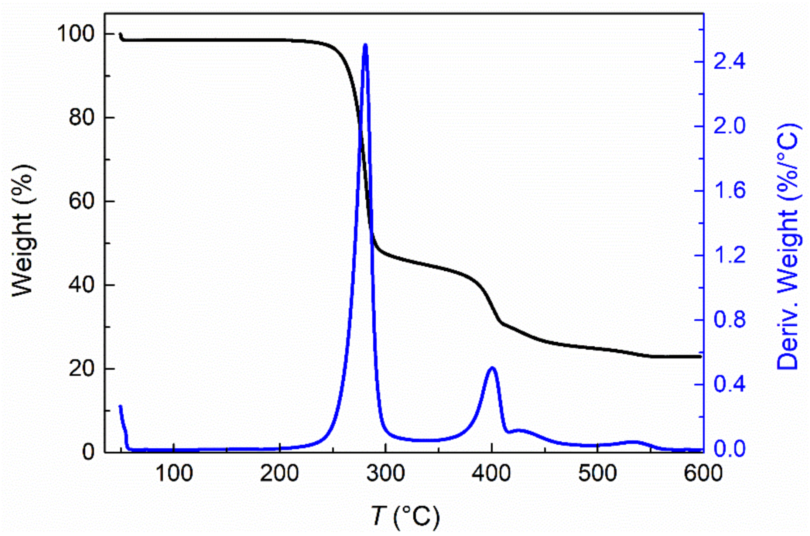

Figure S20. Thermogravimetric analysis of **3** between 30 °C and 600°C.

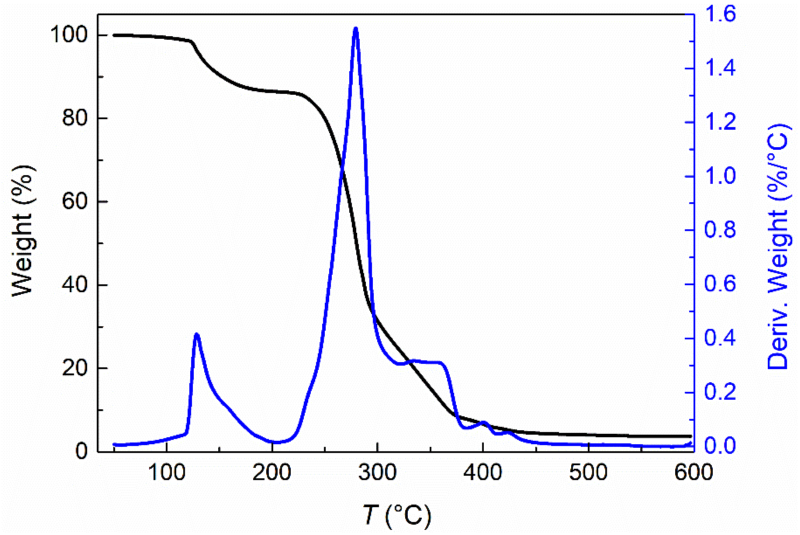

Figure S21. Thermogravimetric analysis of **3@PMMA** between 30 °C and 600°C.

9) Absorption spectra

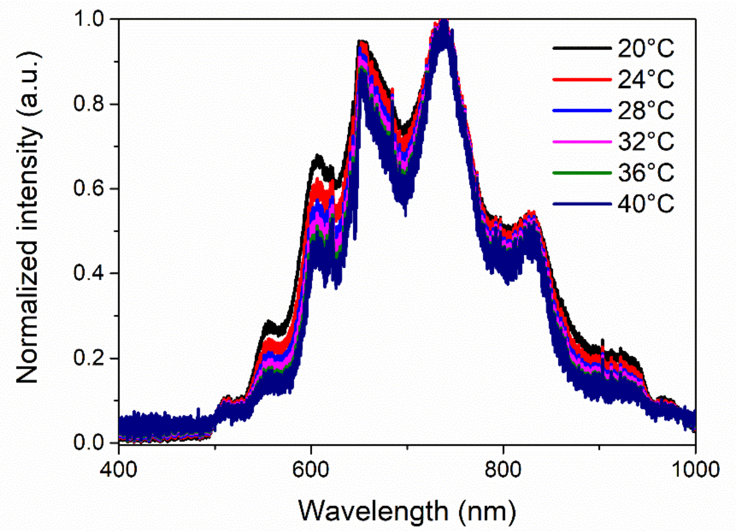

Figure S22. Absorption spectra for **1@PMMA** at different temperatures.

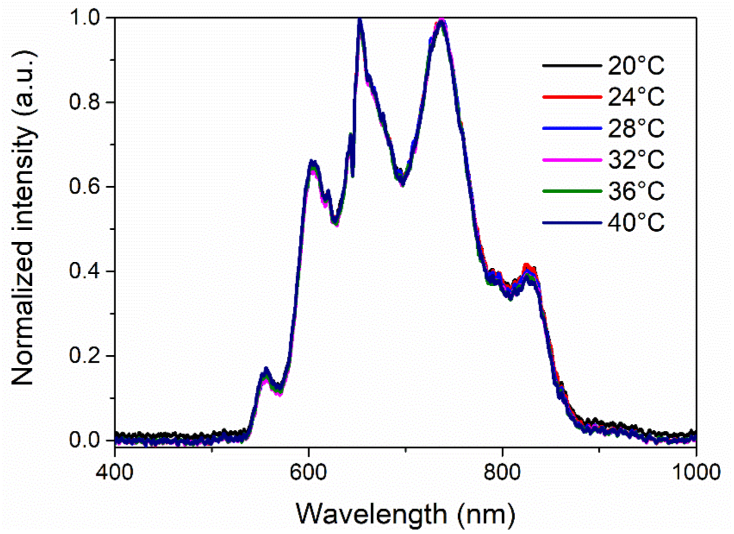

Figure S23. Absorption spectra for **2@PMMA** at different temperatures.

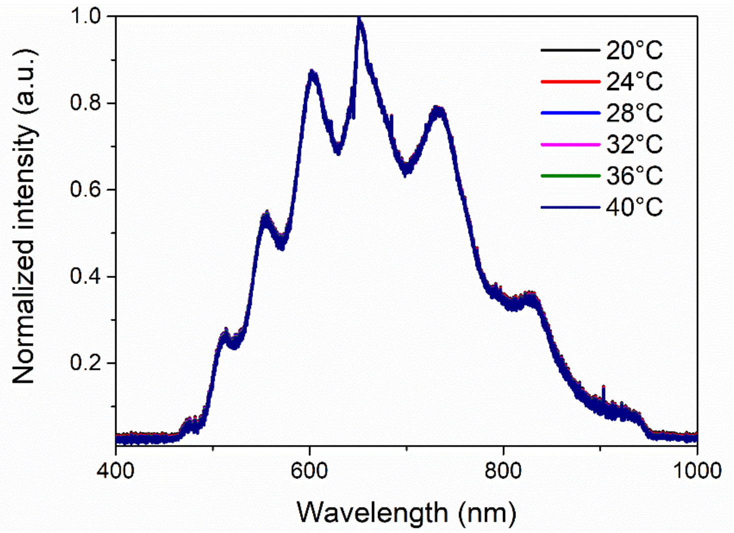

Figure S24. Absorption spectra for **3@PMMA** at different temperatures.

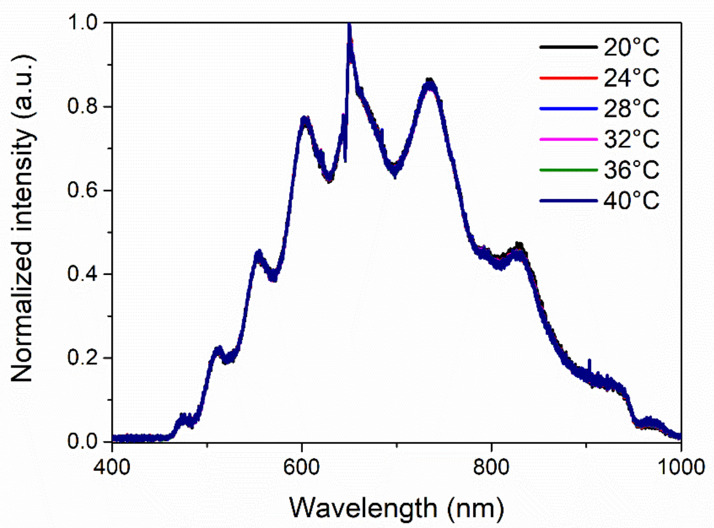

Figure S25. Absorption spectra for PMMA at different temperatures.

10) First experimental setup

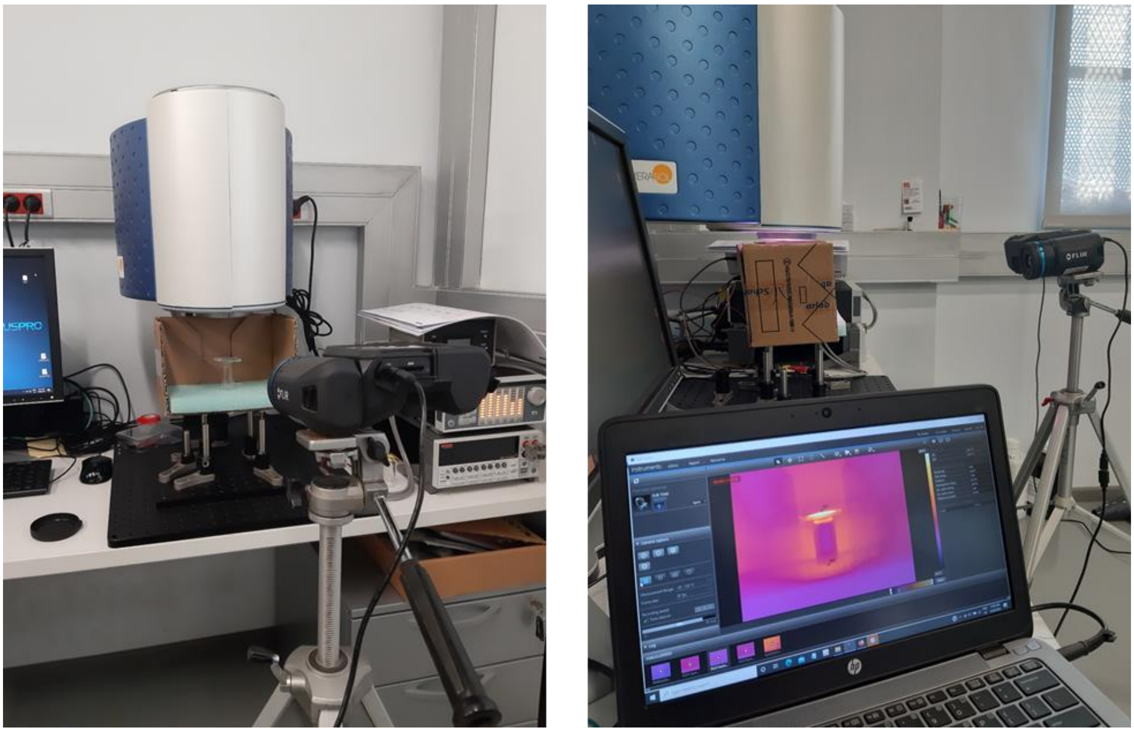

Figure S26. First experimental setup.

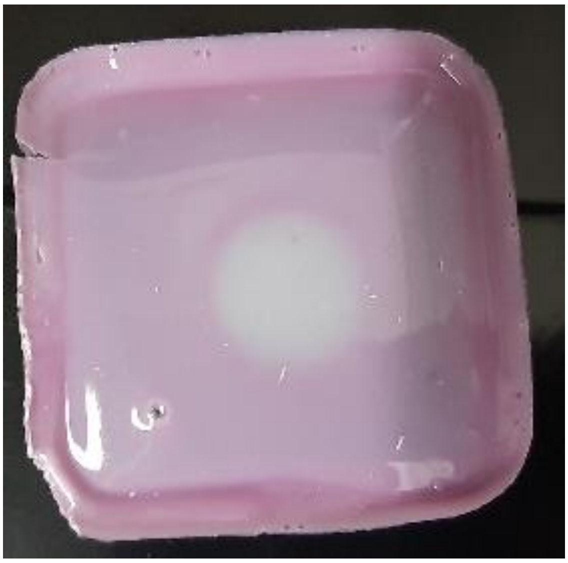

Figure S27. 1@PMMA after exposition to the solar lamp, where the HS and LS phases can be seen by bare-eye.

11) Second experimental setup

The second experimental setup consists on two Peltiers, which are in contact with a copper sheet of 0.5 mm, that acts as a sample holder. A round hole (d = 10 mm) is drilled into this copper foil. On top of this there is another copper sheet with a square window (40 mm x 40 mm) where the sample is placed. Another sheet, equal to the first one, goes on top of the sample, so that it is sandwiched by copper sheets from all directions, except the hole, that allows the light irradiation to pass through the sample. To avoid heating of the copper foils with the solar simulator, a piece of insulating foam is placed on top, covering the copper sheets, except the transmission aperture. Directly below this orifice, there is an anodized aluminum sensor (area 10 mm x 10 mm; distance from sample: 25 mm), surrounded from all sides, except from above, by insulating foam. To eliminate temperature fluctuations coming from the Peltiers, two metal sheets (225 mm x 95 mm x 1 mm) are placed in between them and the sensor, so the temperature flow is perpendicular to the Peltier-sensor-Peltier direction. The temperature is monitored in the sensor by a thermocouple. Right next to the sample there is another thermocouple attached, but to accurately control the sample temperature the thermal camera is used. The solar simulator is placed on top of this setup (distance to the sample 120 mm).

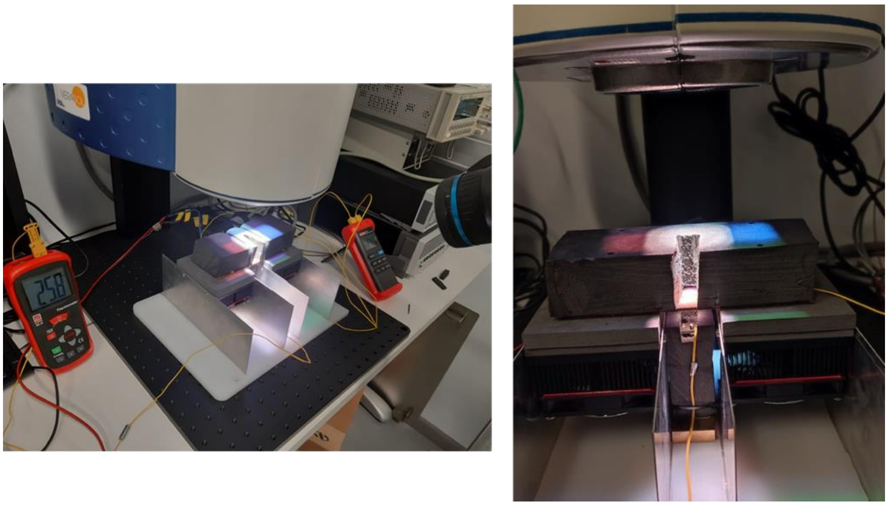

Figure S28. Second experimental setup.

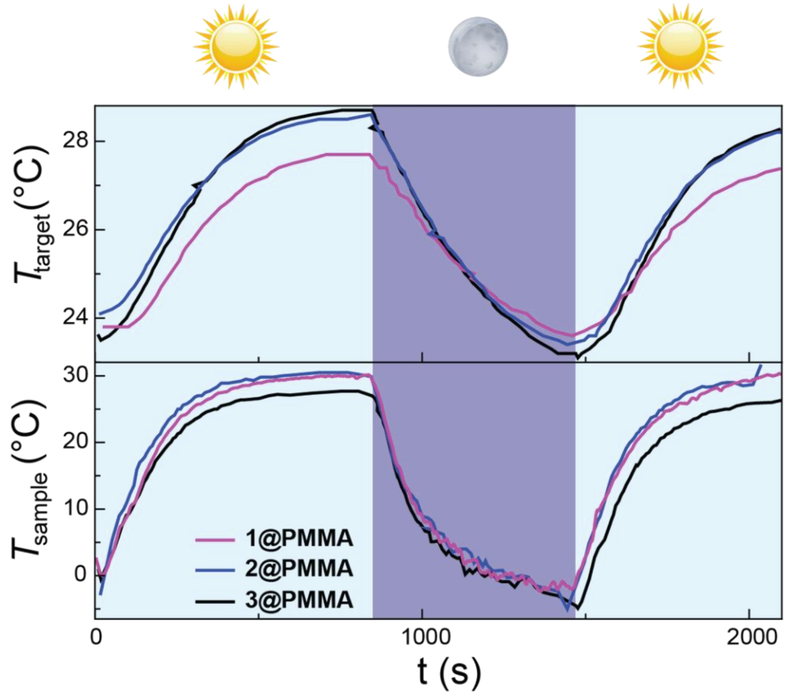

Figure S29. Repetition of the on-off solar simulator cycles experiments.

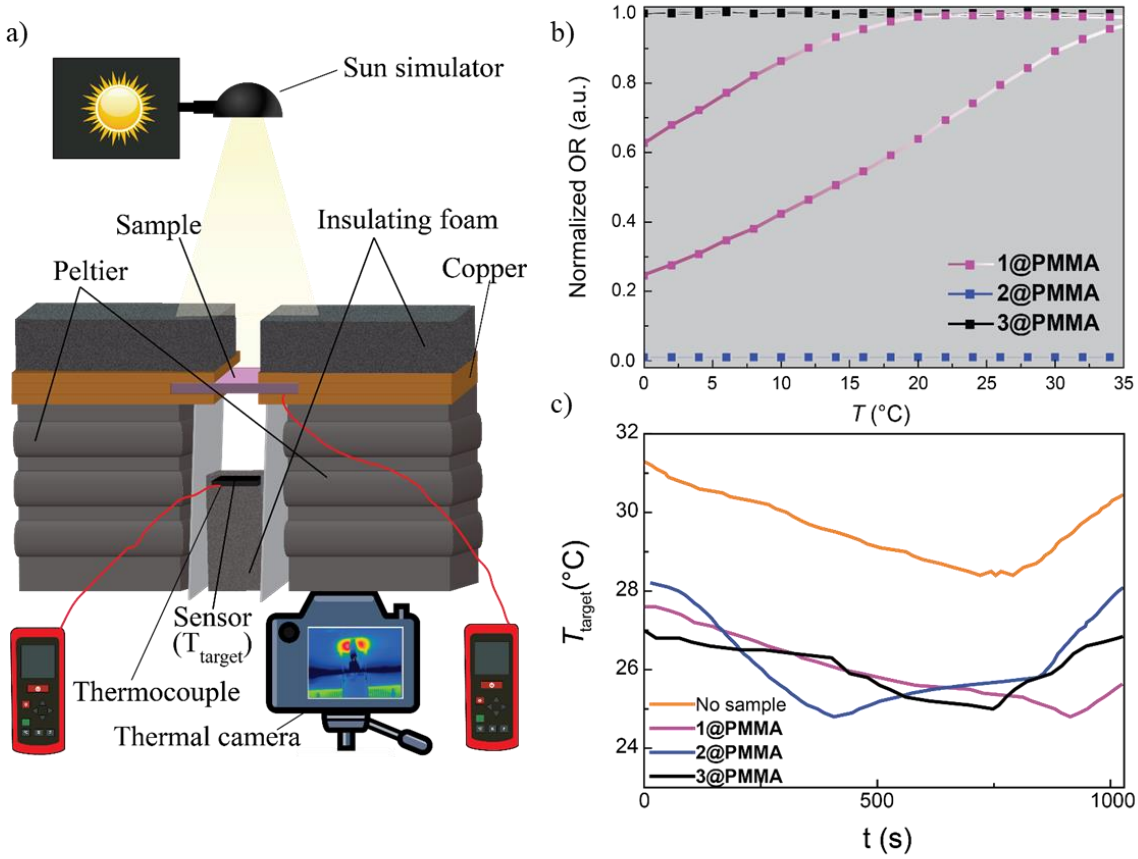

Figure S30. a) Scheme of the experimental setup. b) Optical reflectivity measurements for **1@PMMA-3@PMMA** in the temperature region encompassed by these sunlight exposure measurements. The optical reflectivity has been normalized in respect to **1@PMMA**. c) T_{target} *vs.* exposure time with the solar simulator turned on and on-off Peltier cycles.

12) Enthalpy values of different phase-change materials

Table S1

Phase-change material	ΔH (kJ·kg^{-1})	Reference
1	50	This work
RT25HC	230	[26]
MC28	170	[59]
HDPE	141.8	[60]
Glycerin	198.7	[23]
Paraffin C16	238	
Propyl palmitate	186	
Butyl stearate	140	
Emerest 2325	134	
Lithium chloride ethanolate	188	
Paraffin C17	213	
RT20	172	
D-Lattic acid	184	
MICRONAL 5001	110	
1-dodecanol	200	
Octadecyl thioglyate	90	
Paraffin C18	244	
Methyl palmitate	205	
ERMEST2325	138	
